# Addressing the Question of Disorder-Specific Risk Factors of Internet Addiction: A Comparison of Personality Traits in Patients with Addictive Behaviors and Comorbid Internet Addiction

**DOI:** 10.1155/2013/546342

**Published:** 2013-06-25

**Authors:** K. W. Müller, A. Koch, U. Dickenhorst, M. E. Beutel, E. Duven, K. Wölfling

**Affiliations:** ^1^Department of Psychosomatic Medicine and Psychotherapy, University Medical Centre, Johannes Gutenberg University Mainz, Untere Zahlbacher Straße 8, 55131 Mainz, Germany; ^2^Bundesverband für Stationäre Suchtkrankenhilfe e.V.(buss), Wilhelmshöher Allee 273, 34131 Kassel, Germany; ^3^Therapeutic Giudance of LWL Rehabilitation Centre Ostwestfalen, Bernhard-Salzmann-Klinik, Buxelstraße 50, 33334 Gütersloh, Germany

## Abstract

Uncontrolled use of the internet has been reported to affect the lives of some users in a negative way. According to epidemiological studies, about 1% of the general population is showing signs of internet addiction. Since internet addiction is becoming a growing health concern, research on potential risk factors is becoming more important in order to develop strategies for prevention and to adopt therapeutic treatment. Although there are some studies investigating personality traits in internet addiction, most of these studies are based on samples of healthy subjects. In this research project, we compared personality profiles of a sample of patients in different rehabilitation centers. 70 patients with an addiction disorder that additionally met the criteria for internet addiction were compared to 48 patients suffering from alcohol dependence. Besides Big Five personality traits, we also assessed depressive symptoms. It was shown that patients with comorbid internet addiction can be discriminated from other patients by higher neuroticism and lower extraversion as well as lower conscientiousness. After controlling for depressive symptoms, lower conscientiousness especially turned out to be a disorder-specific risk factor. As internet addiction is related to unique patterns of personality traits and can be discriminated from alcohol dependence, treatment approaches are needed that meet the specific requirements of patients with internet addiction.

## 1. Introduction

Since its development in the 1990s and rapid dissemination in the years thereafter, the internet has become one major part of our daily lives. However, epidemiological studies and clinical reports indicate that internet behavior sometimes can become excessive and uncontrolled and thereby affect the lives of people in a rather negative way. An early study by Kraut et al. [1] found that in some internet users psychological wellbeing was deteriorating while the time spent online became more and more excessive. Likewise, Young [2] provided first clinical reports of patients showing patterns of abnormal internet usage behavior that she called internet addiction (IA). 

15 years later, the American Psychiatric Association (APA) decided to include “Internet Gaming Disorder” as a preliminary diagnosis in section III of the DSM-V [3, 4]. This decision was based on a comparably large scientific and clinical literature dealing with the phenomenon of addictive internet use sometimes referred to as “problematic internet use” [5], “pathological internet use” [6], or “internet addiction” [2, 7]. Despite the different labeling there are increasing scientific contributions referring to the term internet addiction (IA). This is mainly due to neuroscientific evidence [8–10] indicating parallels between IA and substance-related addiction disorders and pathological gambling. To no surprise, proposed diagnostic criteria for IA (craving, tolerance, and withdrawal) resemble those of other addictive behaviors [7, 11–13]. 

International epidemiological studies emphasize that IA is a cause of public health concern with prevalence rates ranging between 0.6 to 1.0% [14, 15] and even increased rates (3–13%) among adolescents [15–17]. Also, IA seems to be related to several psychosocial and physical problems, like decreased wellbeing and social skills [18, 19], impaired job performance [20], and increased rates of sleep disorders [21]. Moreover, different investigations come to the conclusion that IA is often related to further mental disorders, especially depressive and anxiety disorders [22].

High levels of psychopathological symptoms and a decreased level of functioning [13] in patients suffering from IA require the development of specific treatment programs. Although we are far from being able to offer sufficient and tailored treatment yet, there are some case reports [11] and even clinical trials [23] published proposing disorder specific treatment programs and first data on efficacy of psychotherapy and use of medication [24].

However, there are still uncertainties regarding the predisposing factors for IA. Although there are some studies existing, most of the findings are based on epidemiological surveys including convenience samples. For example, Ko et al. [25] found that reward dependence was decreased and novelty seeking was increased in IA among Asian students with IA. Also Rahmani and Lavasani [26] found that those suffering from IA in a student sample reported decreased scores in conscientiousness and agreeableness according to the Five-Factor Model of personality [27]. In a similar survey Kuss et al. [28] identified increased neuroticism and low agreeableness as risk factors for IA.

Investigating patients in treatment because of IA, Dreier et al. [29] found that those patients showed distinct patterns regarding Big Five personality traits compared to clinical control subjects. In detail, IA was associated with heightened neuroticism and decreased conscientiousness. To a lesser extent, extraversion was also diminished. Based on these data, the authors [29] proposed an etiopathological model of IA [30, 31], assuming that a unique constellation of personality traits (increased neuroticism, decreased conscientiousness, and extraversion) can lead to conflicts between an individual and his social environment and foster signs of withdrawal into virtual worlds where the individual can feel safe. The basic assumptions of this model have been supported by first clinical findings [31].

As knowledge about predisposing factors is essential for the development of prevention strategies and psychotherapy (e.g., within psychoeducation), as well as for the clinical understanding of IA, we intended to further elucidate this field by a clinical investigation. Moreover, we aimed at evaluating the aspect of disorder specificity of previous findings on personality traits in IA. To that purpose, we recruited a sample of patients from different rehabilitation centers across Germany. Each patient entered treatment because of a substance-related addiction disorder or pathological gambling and was screened for IA. As reported elsewhere [32], we found that 4.2% of this sample met the criteria of comorbid IA. In a second wave, we recruited patients suffering from alcohol dependence of the same rehabilitation centers not fulfilling the criteria of IA and compared both groups.

We hypothesized that patients with comorbid IA can be discriminated from the control group on the basis of personality traits according to the Five Factor Model. In detail, we expected to find increased neuroticism in patients with comorbid IA, as well as low conscientiousness and low extraversion.

In order to control for the effects of depression that have been shown to affect personality variables independently [33] and that have been reported to be common among patients with IA, we used the Beck Depression Inventory (BDI-II; [34]) to screen for depressive symptoms.

## 2. Materials and Methods

### 2.1. Recruitment and Description of Sociodemographics of the Sample

15 inpatient rehabilitation centers of the “German Federal Association of Inpatient Addiction Rehabilitation” (Bundesverband für Stationäre Suchtkrankenhilfe, “buss”) served as recruitment centers. Every consecutively patient entering treatment within a time frame of six months was informed about the research project and was asked for written informed consent. Participation did not affect treatment and was voluntary. The declaration of Helsinki was preserved. 

In wave 1 a total of 1826 patients were screened for IA and additional variables (e.g., personality traits, depression; see Section 2.3 for a description of the inventories). 77 patients met criteria for comorbid IA (comorbid IA-group). Among these patients the most common primary diagnoses were alcohol dependence (41.5%), pathological gambling (27.7%) and dependence on cannabinoids (26.2%). 7 patients of this group had to be excluded from further analyses, one because of female gender and six because of missing data. The final comorbid IA group consisted of *N* = 71 male patients. In wave 2 a supplementary recruitment among those patients who did not meet criteria was performed. Only patients suffering from alcohol dependence were selected who matched members of the comorbid IA group according to age and gender. Initially a total of 66 patients were successfully recruited for the alcohol group; however, due to missing data, only 48 male patients entered the final statistical analyses. The mean age of the IA group was 29.3 years (SD = 10.66; range 16–64) and did not differ from the alcohol group (31.7 years; SD = 9.18; range 17–65; ns). The project was funded by the German federal Ministry of Health (Grant no. IIA5-2509DSM119).

### 2.2. Statistical Analyses

All the analyses were performed using SPSS 21.0 (SPSS Inc, Chicago, IL, USA). Simple group differences were calculated with *t*-tests. Chi-square tests and Fisher's Exact tests were applied for comparisons of dichotomous variables with the coefficient Cramer-V as a measure of effect size. Analyses of variance (ANCOVA) were performed to analyze detailed subgroup differences. Pearson's *r* was calculated to determine correlations between metric variables. *P* values smaller than .05 were considered as indicating significant differences.

### 2.3. Measures

Internet addiction was assessed using the Scale for the Assessment of Internet and Computer game Addiction (AICA-S; [35]). AICA-S is a self-report measure whose items (on 5-point likert scales and in forced choice format) are related to the DSM criteria of substance-dependence (craving, tolerance, withdrawal, and loss of control). Previous studies on its psychometric properties yielded satisfying results concerning item characteristics, reliability, and validity. According to the results of a clinical validation [36] and based on a representative sample [37], a score of 7 points leads to the best diagnostic accuracy in dividing addictive from healthy internet use.

The NEO Five Factors Inventory (NEO-FFI; [27]; German version [38]) consists of 60 items answered in 5-point Likert-type scales (0 = strongly disagree to 4 = strongly agree). It is one of the most widely used questionnaires for measuring the Big Five and has been repeatedly demonstrated to have good psychometric properties.

The BDI-II [34] is a 21-item self-report assessing depressive symptoms. A sample of symptoms that are depicted by the items includes sad mood, pessimism, loss of pleasure, self-blaming, loss of energy, and desire to commit suicide. The answer options reflect four severity levels of depression (on 4-point likert scales). Item responses are summed and display the severity of a depression with the following cutoffs: 0–13 (no depression), 14–24 (moderate depression), and more than 24 points (severe depression).

Studies using the scale indicate that it is an appropriate method for assessing signs and levels of depression. The BDI-II has been demonstrated to have good psychometric properties in clinical and nonclinical samples with Cronbach's Alpha of .92 and retest reliability of .93. Its construct validity has been proven as well (*r* = .93) and there are moderate to high correlations between the BDI-II and validity-convergent scales.

## 3. Results

### 3.1. Personality Traits

To test if there are systematic differences in the Big Five between the clinical groups, simple *t*-tests were calculated. It turned out that significant differences in three factors were observable. As can be derived from Table 1, patients with comorbid IA showed higher neuroticism and lower extraversion and conscientiousness than the alcohol group.

### 3.2. Depressive Symptoms and Personality Traits

Based on the BDI-II, we found 33.9% (40) of the total sample showing no signs of depression, 25.4% (30) exceeding the cutoff for mild depressive symptoms, and further 40.7% (48) scoring above 24 points and therefore being classified as suffering from severe depressive symptoms. In a further analysis it was tested whether depressive symptoms were more prominent among patients with comorbid IA than among those suffering from alcohol dependence. A *t*-test (*t*(115) = 4.248; *P* ≤ .001) revealed that the comorbid IA group (M = 20.69, SD = 12.74) scored significantly higher in BDI-II than the alcohol group (M = 12.08, SD = 9.25). Also, a chi-square test yielded a significant result (*χ* ^2^(2) = 9.668; *P* < .001; Cramer-V = .29) indicating that the comorbid IA group (51.4%; *n* = 36) showed more signs of severe depression than the alcohol group (25.0%; *n* = 12). We also analyzed possible relationships between depressive symptoms and personality traits using correlational analyses between the scores in the BDI-II and the NEO-FFI scales. It turned out that there were significant correlations between depressive symptoms and neuroticism (*r* = .70; *P* ≤ .01), extraversion (*r* = −.49; *P* ≤ .01), agreeableness (*r* = −.24; *P* ≤ .05), and conscientiousness (*r* = −.36; *P* ≤ .01).

**Figure 1:**
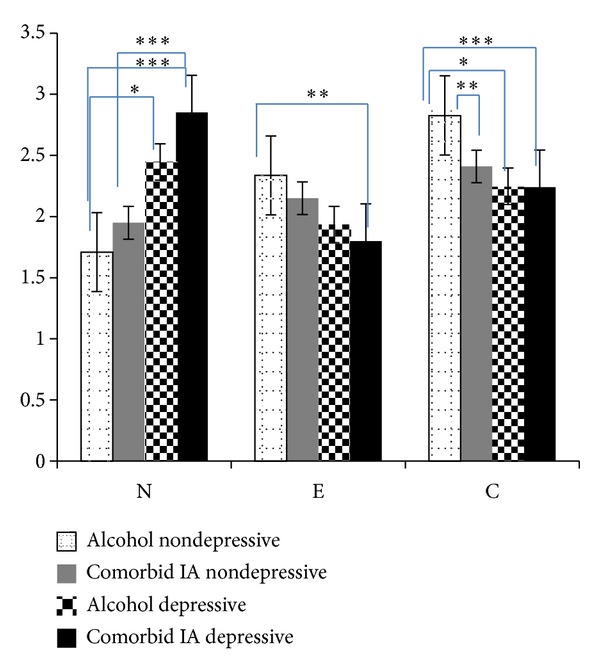
Means and standard errors of neuroticism, extraversion and conscientiousness according to internet addiction, alcoholism and associated severity of depressive symptoms. Comments: *N* = 118; N = neuroticism; E = extraversion; C = conscientiousness; means of each personality trait are depicted on the *y*-axis; level of significance: *P ≤ .05; **P ≤ .01, ***P ≤ .001.

Finally, we intended to analyze the coexisting influence of depression on personality traits in the two clinical groups. We therefore calculated the following groups: patients with comorbid IA that exceed the BDI cutoff of 24 points (comorbid IA depressive; *n* = 36), patients with comorbid IA scoring below the BDI cutoff of 24 points (comorbid IA nondepressive; *n* = 34), patients without IA scoring higher than 24 points in the BDI (alcohol depressive; *n* = 12), and patients without IA scoring below 24 points in the BDI (alcohol nondepressive; *n* = 36). We than compared those 4 groups according to their personality traits by conducting a multiple ANOVA that yielded significant main effects only for neuroticism (*F*(3) = 31.183, *P* ≤ .001), extraversion (*F*(3) = 5.829, *P* ≤ .001), and conscientiousness (*F*(3) = 8.636, *P* ≤ .001). Dunnett's *t*_3_-tests were performed as post hoc analyses in order to elucidate detailed differences between the 4 groups. The results are depicted in Figure 1. 

As can be derived from Figure 1, presence of severe depressive symptoms is adding information to the associations between personality and IA reported previously. In particular, neuroticism seems to vary taking depressive symptoms into account. Patients with both comorbid IA and signs of depression as well as alcoholics with depression express the highest scores in neuroticism, while there are no differences remaining between subjects with comorbid IA and the alcoholics when no depressive symptoms are involved. Regarding extraversion comorbid IA patients with depression can be contrasted against nondepressive alcoholics (*P* ≤ .01) but there are no further differences between the groups. Regardless of presence of depressive symptoms, patients with comorbid IA show significantly diminished scores in conscientiousness compared to nondepressive alcoholics. However, none of the comorbid IA-groups show significant differences in any of the three personality factors compared to alcoholics with depression.

## 4. Discussion

In this study, we assessed personality traits according to the model of the Big Five and depressive symptoms in relation to comorbid internet addiction among 118 patients in treatment for addiction disorders. Our research aimed at identifying premorbid patterns of personality of patients with comorbid internet addiction. In previous research on treatment seekers with internet addiction, it was shown that heightened neuroticism along with decreased extraversion and conscientiousness was characteristic for these patents [30, 31]. Also, first large-scale epidemiological surveys gave support to this assumption [28, 39, 40], although a few studies came to different conclusions [41].

In order to better understand potential antecedents of internet addiction we tried to discriminate personality traits not solely by focusing on patients with internet addictive behavior, but within a clinical sample of patients with a different addiction disorder who additionally met criteria of internet addiction. Patients with alcohol dependence served as a control group. As internet addiction has been reported to often cooccur with depressive disorders, we also determined frequency and severity of depressive symptoms within these patients and consecutively analyzed potential effects on personality.

As expected, we found specific differences in personality traits between patients with and without comorbid internet addiction. Patients with comorbid internet addiction displayed increased neuroticism compared to patients suffering from alcohol dependence. Generally, neuroticism is regarded as a global risk factor for health. Different studies have demonstrated that elevated neuroticism is related to higher rates of psychosomatic complaints and psychopathological symptoms [42, 43]. Also, clinical investigations have shown that heightened neuroticism is associated with an increased risk for generalized anxiety disorder, panic attacks, and depressive disorders [44], as well as pathological gambling [45]—a clinical disorder closely related to internet addiction. One explanation for increased neuroticism in internet addicts can be supposed in negative emotional states and weak self-esteem related to neuroticism (e.g., [46]). Feeling moody and dysphoric in real-life contexts might motivate the individual to search for new areas where he can avoid his depressed feelings. Virtual environments might have the potential to be regarded by this individual as safe and comfortable surroundings that offer a huge amount of distractions (e.g., games, chats, research portals, and music). So, for this person, the internet might become even more attractive than to a person with high emotional stability. By avoiding stressors of the everyday life the vulnerable individual will experience negative reinforcement and hereby spending increasing time online—becoming bound to the internet.

Also we found patients with comorbid internet addiction to express lower scores in extraversion in comparison to patients with alcohol addiction. Decreased extraversion has been identified as a risk factor for a variety of mental disorders, including major depression [44]. In the same time increased extraversion has been reported to be associated with heightened risk for drinking alcohol and smoking [47]. However, based on case reports, clinical experience and epidemiological surveys [31], we expected to find rather lower than higher scores in extraversion in internet addicts, and this assumption was supported by our data. As low extraversion has been demonstrated to be related to social detachment and social insecurity, the communicative possibilities provided by the internet and online communication can be perceived as an additional attractor for introverted individuals. The introverted user might experience the desire to be part of a social network but on the same time is feeling uncomfortable in face to face contacts. (Mis-) using online communication as a replacement for real-life interactions might be regarded as further negative reinforcement for these patients. 

Finally, significantly lower scores in conscientiousness were found within the group of internet addicts. Low conscientiousness has been linked to poorer achievement-orientation and to being unstructured and disorganized. Persons oblivious to real-life duties might be at greater risk losing themselves in the endless virtual worlds and being more easily distracted by the possibilities of the internet. Furthermore, similar to heightened neuroticism and decreased extraversion, low conscientiousness has been demonstrated to predict health problems [48, 49].

In correspondence to other reports [22] we found high rates of severe depressive symptoms in half of the patients expressing comorbid internet addiction. Based on the classification of the BDI-II we investigated in how far basic personality traits were additionally influenced by severe depressive symptoms. It turned out that all of the three personality domains were affected by depressive symptoms. Patients with internet addiction and depression revealed highest scores in neuroticism, with significant differences also between internet addicts with and without depression. No differences were found between nondepressive internet addicts and the alcohol group. Taking depressive symptoms into account, the effects of extraversion were reduced and remained present in internet addicts with depression that differed significantly in contrast to nondepressive alcoholics. Conscientiousness was unaffected by depressive symptoms in the group of internet addicts compared to nondepressive alcoholics. This indicates that especially the factor conscientiousness may play a key role as a disorder-specific risk factor for internet addiction.

There are several possible explanations for the effects of depressive symptoms on personality. One explanation is that the combination of internet addiction and depressive symptoms is to be regarded as a special subtype of internet addiction with specific trait constellations that might turn out to be different from general etiopathological models. As internet addiction is believed to be a heterogeneous construct, [20] it sounds reasonable that specific subgroups within patients are present. An alternative explanation might be seen in confounding effects that could have led to a data bias. As depressive patients display a number of biases concerning perception on their selves and in cognition, it might be the case that the differences found in the present study are due to known shortcomings of self-report measures that require a certain amount of objective self-reflectiveness. As depressive subjects underlie distortions in self-perception and tend to experience their personal situation as rather desolate, helpless, and hopeless, it might have led to confounding effects. One possibility to test this hypothesis is to apply measures of personality that are less related to objective self-reflectiveness, for example implicit measures based on reaction times [50]. However, it might also be the case that effects of the depressive episode itself have caused deviances in the personality profiles. In a follow-up investigation of depressive patients in treatment, Costa and colleagues [33] found that there were changes in personality before and after treatment. The authors interpret their findings as being due to the remission of depressive symptoms that were responsible for an alteration of personality traits, especially affecting neuroticism, extraversion, and conscientiousness. This notion is supported by an early investigation by Wasek and Endicott [51] who also found differences in personality profiles of depressive subjects depending on experiencing acute depressive symptoms or not.

As the current study used a cross-sectional design and was based on a small sample size, generalizability of its findings is strictly limited, and it is not possible to draw causal conclusions from the data. Moreover, due to the small sample size, it was not possible to further divide the heterogeneous group of patients with comorbid IA according to their primary diagnoses (e.g., alcohol dependence, pathological gambling) and perform further analyses on personality traits. It is necessary to further elucidate the role of personality and its disorder specificity for internet addiction in upcoming research projects based on larger sample sizes. Up to now, the current study should be regarded as a first contribution indicating that internet addiction can be characterized by specific personality traits that might be influenced by further psychopathological symptoms.

## 5. Conclusion

Systematic differences according to the Big Five personality traits were identified between patients suffering from an addiction disorder and patients showing internet addictive behavior as a comorbid disorder. In detail, we found internet addiction to be associated with heightened neuroticism and decreased extraversion and conscientiousness, supporting findings on premorbid personality factors of previous studies [28, 31]. Also, we found high rates of severe depressive symptoms within patients suffering from comorbid internet addiction. It also turned out that depressive symptoms had an independent influence on personality traits, especially on neuroticism. As there are personality-related differences between patients with comorbid internet addiction and patients suffering from other kinds of addiction, it seems insufficient to treat internet addiction in exactly the same way as other addictive behaviors. Adopted treatment approaches are needed to meet the disorder-specific demands of patients affected by internet addiction.

## Figures and Tables

**Table 1:** The Big Five in comparison between patients with and without comorbid internet addiction (means and statistical significance).

Domains of the NEO-FFI	Total (N = 118) M (SD)	Comorbid IA group (N = 70) M (SD)	Alcohol group (N = 48) M (SD)	Level of significance
Neuroticism	2.20 (.71)	2.41 (.69)	1.89 (.64)	*t*(116) = 4.149; *P* ≤ .001
Extraversion	2.08 (.60)	1.97 (.66)	2.24 (.46)	*t*(115) = 2.577; *P* ≤ .05
Openness	2.27 (.60)	2.22 (.58)	2.36 (.61)	*t*(116) = 1.256; ns
Agreeableness	2.14 (.32)	2.11 (.33)	2.19 (.29)	*t*(116) = 1.386; ns
Conscientiousness	2.47 (.58)	2.32 (.57)	2.68 (.52)	*t*(116) = 3.479; *P* ≤ .001

Comments: M: mean; SD: standard deviation.
